# Factors Associated With Body Weight Status of Iranian Postgraduate Students in University of Putra Malaysia

**DOI:** 10.5812/nms.9186

**Published:** 2013-12-10

**Authors:** Maryam Zarei, Mohd Nasir Mohd Taib, Fatemeh Zarei, Hazizi Abu Saad

**Affiliations:** 1 Department of Nutrition, Ministry of Health and Education, Tehran, IR Iran; 2 Department of Nutrition, University of Putra Malaysia, Kuala Lumpur, Malaysia; 3 Food and Drug Organization, Ministry of Health and Education, Tehran, IR Iran

**Keywords:** Obesity, Life Style, Diet Records, Malaysia, Students

## Abstract

**Background:**

Good nutrition, a balanced diet and regular physical activity are foundations of good health. Research has found that dietary patterns change dramatically following the arrival of students in a foreign country. However, nutritional status of Iranian students studying oversea has never been investigated.

**Objectives:**

The objective of this study was to determine factors associated with body weight status of Iranian postgraduate students in Universiti of Putra Malaysia (UPM).

**Materials and Methods:**

A cross-sectional study was conducted to determine the body weight status of 210 Iranian postgraduate students aged between 22 and 55 years in University of Putra Malaysia. The associations between body weight status and socio demographics factors and also lifestyle factors (smoking and physical activity) were assessed. Anthropometric factors (height, weight, BMI and waist and hip circumferences) were measured. Chi-square, Spearman Rho and Pearson tests were used for data analysis.

**Results:**

From a total of 210 postgraduate students 110 were females, and 100 males. No significant correlation was observed between smoking and BMI (P = 0.4). However, statistically significant correlations were observed between gender (P = 0.007), physical activity (P = 0.02), using protein (P = 0.005), carbohydrate (P = 0.002), fat (P = 0.001), fiber (P = 0.003), vitamin C (P = 0.04), calcium (P = 0.005), waist circumference (P = 0.02), hip circumference (P = 0.001), Waist to Hip Ratio (P = 0.002), and BMI.

**Conclusions:**

The nutritional behavior of university students was poor. Therefore, it is essential to encourage young people, including university students to enrich their diets with milk, beans, fruit, and vegetables to decrease the risks of nutrition related disorders.

## 1. Background

Nutrition is an input to and base for health and development. Good nutrition, a balanced diet and regular physical activity are foundations of good health. Good nutrition is the cornerstone for strong immune system, disease prevention, and a better health (1). The prevalence of obesity has been increased dramatically worldwide. Recent estimates suggest that a Body Mass Index (BMI) over 25 kg/m^2^ is responsible for 64% of male and 77% of female cases of type 2 diabetes mellitus worldwide (2). Whilst students leave home to attend college, their nutrition and physical activity become their exclusive responsibility. During the rise of a student to independence, they seek modest or no guidance from adults as to their health or dietary choices (3). Then, changes would occur in their food consumption patterns which is characterized by decreased milk products and fruit consumption, and increased alcohol and sweetened beverage consumption (4). A student’s lifestyle may also change notably in a foreign country. Research has found that dietary patterns change dramatically following the arrival of students in a foreign country, and students are at risk of developing unhealthy eating habits, especially during their first year of inhabitancy (5). Poor dietary habits and health behaviors among university students have been reported in different countries such as Armenia and the United States (6, 7). Smoking and physical inactivity are risk factors for several diseases. In most developed countries the trends in many health behaviors are not changing toward a healthier direction. Preventing risky behaviors in adolescence is important, because behaviors are being formed, and then would affect health and health behaviors in adulthood (8). There are, however, exceptions to this rule: a number of people with an “obese” BMI may have acceptable amount of body fat merged with a large muscle mass; whereas, other individuals with a “normal” BMI may have severe adiposity and reduced muscle mass (9). Individual assessment of nutritional status of population groups has a vital significance for public health. In this respect, the nutritional status of adults is demonstrated by BMI which is the result of dividing weight (in kilograms) by the square of the height in meters (kg/m^2^). According to the World Health Organization, there are four major categories for adults BMI including: underweight (< 18.5 kg/m^2^), healthy weight (18.5 - 24.9 kg/m^2^), overweight (25 - 29.9 kg/m^2^), and obese (> 30 kg/m^2^) (10). The purpose of the present study was to determine factors associated with body weight status among young adults. Many studies have reported that body weight status is associated with socio-demographic factors such as sex, age, marital status, and living arrangements (11-13). Unfavorable nutritional outcomes, such as obesity and chronic diseases, resulting from migration or the mobility of population groups have been reported recently by several authors (14-16). Eating behaviors, low quality diet, and physical inactivity may result in a greater risk of obesity in adulthood (4). An unpublished study in Malaysia has reported that the prevalence of overweight and obesity among university students was typically due to their low level physical activity (17). The number of foreign students studying in Malaysia is increasing dramatically since 2003. Currently, approximately 4000 foreign students study in University of Putra Malaysia (UPM). However, nutritional status of foreign students and especially Iranian students studying at higher educational institutions such as UPM has never been investigated. Therefore, it is crucial to study factors affecting body weight of Iranian postgraduate students in UPM.

## 2. Objectives

The objective of this study was to assess factors associated with BMI of Iranian postgraduate students in UPM, Malaysia.

## 3. Materials and Methods

A cross-sectional study was conducted at UPM, in Malaysia. UPM is a destination for a large number of international students, including Iranian students. The minimum sample size was calculated based on a previous study, showing a prevalence of 0.124 for obesity among university students (18). Then 168 samples were estimated to be needed based on the following parameters (α = 0.05, β = 0.80, P = 0.124, and sampling error of 0.05). However, the sample size was increased by 30%, and finally 210 samples were selected (from a total of 1458 Iranian postgraduate students in 2009) to compensate possible attritions. To achieve the needed sample size, the researcher obtained a list of Iranian postgraduate students from the center of Student Graduate Studies. The list included the students in 15 faculties and seven institutes of UPM. The instrument used in this study was a self-administered questionnaire divided into three major sections. The first section was consisted of seven questions about socio-demographic factors including the student’s gender, age, academic semester in college, program of study, marital status, living arrangement, and residency. In the second section, lifestyle factors were assessed by questioning about smoking and physical activity. The respondents were asked whether they ever tried to smoke even one or two puffs. If the answer was “Yes”, the respondent was required to answer a question on initial age of smoking. The participants were asked to recall their physical activity for the past seven days prior to the study, regarding the frequency (days per week) and duration (time per day) for each specific type of activity. The level of physical activity was then calculated using the guidelines for data processing and analysis of the international physical activity questionnaire (19). Two types of scores were calculated: continuous score and categorical score. Continuous score was expressed as a MET-minute per week (multiple of resting metabolic rate). A MET-minute was computed by multiplying the MET score by the minutes performed by the three specific types of activity: vigorous-intensity activities, moderate-intensity activities, and walking and days per week. The classification of MET levels of specific types of activity (19) showed that walking = 3.3 METs, moderate = 4 METs, and vigorous= 8 METs. The total MET-min per week was calculated by using the formula as shown below: 

Total MET-minutes per week = (Walking MET × min × days) + (Moderate MET × min × days) + (Vigorous MET × min × days)

Then the total MET-minutes per week were converted into categorical levels. Based on a previous study (20) MET-minutes/week of 0 to 559 was categorized as “low” level physical activity, and then 600 to 1499 and more than 1500 MET-minutes/week were categorized as “moderate” and “high” levels of physical activity, respectively. In the third section, dietary intake of each respondent was assessed trough a 24-hour dietary recall interview. A 24-hour dietary recall for two days (one weekday and one weekend) was used to assess all foods and beverages an individual consumed in the two recent days (midnight to midnight) to calculate respondents’ intake of macro- and micronutrients. Foods consumed were converted into weights in grams based on a list of food weights according to the Food Composition Table (21). Food quantities and the weight of the foods consumed were converted and entered into Nutritionist ProTM for analysis. Obtained data was analyzed based on the Malaysian food database using Nutritionist ProTM software (First Data Bank). If cooked dishes like certain Iranian food were not included in the database of Nutritionist Pro™ Software, the investigator made a recipe for each dish (per 100 gram), and then the quantitative information was entered to Nutritionist Pro software. A 24-hour dietary recall was used for statistical analysis. Mean intakes of nutrients were compared to the Dietary Reference Intake (DRI). The system is used by both the United States and Canada, and is intended for the general public and health professionals. For energy, protein, carbohydrate, vitamins C and minerals (calcium) adequacy was considered achieved if the individuals’ mean intake exceeded 100% of the DRI. Dietary Reference Intake has all macronutrients except fat which is not determined (ND). Anthropometric measurement of height and weight was performed by the researcher. BMI was calculated by dividing body weight in kilograms by the square of their height in meters. Waist circumference was obtained by positioning a measuring tape around the abdomen at the highest lateral border of the right iliac crest. The hip circumference was measured at the point where the buttocks extended the maximum, when viewed from the side. Two consecutive recordings were made for each site to the nearest 0.5 cm on a horizontal plane without compression of skin. Waist and hip circumferences were used to calculate the Waist to Hip Ratio (WHR), in which the waist circumference (cm) is divided by the hip circumference (cm). According to Edmonds et al. (11), waist circumference and WHR were classified into at risk and normal. Pretesting for the questionnaire was performed among 20 Iranian students who were not included in data collection on the first of June 2009 in UPM. The pretest was conducted to ensure that the items and questions in the questionnaire can be understood. The researcher took about 15 minutes to explain the purpose of the study to each participant and measured all students’ body weight; height, hip and waist circumferences. Then the respondents took between 40 - 50 minutes to answer the questionnaire. The pretest confirmed that the respondents could understand most of the questions. Therefore, some modifications were performed in the questionnaire. The reliability of pretested questionnaire was measured by Cronbach’s Alpha; 0.71. 

### 3.1. Ethical Considerations

This study was approved by the Medical Research Ethics Committee of Faculty of Medicine and Health Sciences of UPM. Aims of the study were explained to the subjects, and they were assured of the confidentiality of personal information before starting the study, and all signed a written informed consent. The researchers observed all ethical issues in accordance with the Helsinki Convention.

### 3.2. Data Analysis 

Descriptive statistics such as frequencies, mean, standard deviation and percentage were calculated for all demographic variables. Chi-square test was used to determine the associations between categorical variables. For parametric variable Pearson product-moment coloration was used to determine the association between two continuous variables. P < 0.05 was considered as statistically significant. 

## 4. Results

A total of 210 postgraduate Iranian students including 110 females (52.4%) and 100 males (47.6%) with a mean age of 30.6 ± 5.57 years were recruited in this study. Also, 109 (51.9%) respondents (51.9%) were Master, and 101 (48.1%) were PhD students. More than a half of the respondents (66.7%) were single, and the others were married. A total of 48 (22.9%) respondents lived on campus. Regarding living arrangements, 69 respondents (32.9%) lived alone, 77 (36.6%) with family, and 64 (30.5%) with their friends. Moreover, 57 (27.1%) students were studying in nursing, medicine, and diet technology, and 153 (72.9%) students were studying in other majors such as agriculture, forestry, and economy. There was a significant positive association between BMI categories and gender (P = 0.02) (Table 1). 

**Table 1.** Association BetweenFactors and Body Weight Status (n = 210)

Variable	Body Mass Index				χ^2^	P value
	Underweight	Normal	Overweight	Obese		
**Gender, No. (%)**						
Female (n = 110)	12 (10.9)	75 (68.2)	16 (14.5)	7 (6.4)	3.78	0.02
Male (n = 100)	6 (6.0)	53 (53.0)	36 (36.0)	5 (5.0)		
**Marital status, No. (%)**						
Single (n = 140)	14 (9.9)	90 (63.8)	30 (21.3)	6 (5)	5.15	0.16
Married (n = 70)	4 (5.8)	38 (55.1)	22 (31.9)	6 (7.2)		
**Residency, No. (%)**						
On campus (n = 48)	5 (9.8)	34 (66.7)	8 (15.7)	4 (7.8)	3.25	0.35
Off campus (n = 182)	13 (8.2)	94 (59.1)	44 (27.7)	8 (5)		
**Living arrangement, No. (%)**						
Alone (n = 69)	7 (9.7)	41 (56.9)	17 (23.6)	4 (9.7)	1.16	0.97
Friend (n = 77)	5 (6.6)	47 (61.8)	21 (27.6)	4 (3.9)		
Family (n = 64)	6 (9.7)	40 (64.5)	14 (22.6)	4 (3.2)		
**Tried smoking, No. (%)**						
Yes (n = 64)	6 (9.4)	32 (50.0)	20 (31.2)	6 (9.4)	5.60	0.13
No (n = 146)	12 (8.3)	96 (65.7)	32 (21.9)	6 (4.1)		
**Physical activity, No. (%)**						
Low (n = 69)	7 (9.7)	41 (56.9)	17 (23.6)	4 (9.7)	1.16	0.01
Moderate (n = 77)	5 (6.6)	47 (61.8)	21 (27.6)	4 (3.9)		
High (n = 64)	6 (9.7)	40 (64.5)	14 (22.6)	4 (3.2)		

In total, 146 (69.5%) respondents were nonsmokers, but 64 (30.5%) ones comprising of 46 (46.0%) males and 18 (16.4%) females had tried smoking. Also, one-third of these respondents tried smoking before the age of 19 years. No significant association was observed between BMI categories and smoking (P = 0.4). However, a significant association was observed between BMI and physical activity (P = 0.01) (Table 1). Table 2 shows significant associations between intakes of protein, carbohydrate, fat, fiber, vitamin C, calcium with BMI. The average percentage of daily intake of carbohydrate contributed 49.4% of total calories; whereas, fat and protein contributed 37.3% and 13.3% of total calories, respectively. DRI achievement showed that carbohydrate and protein intakes were high in both sexes which exceeded 100% of DRI. Energy, fiber, vitamin C, calcium and iron intakes were less than the recommended intake in DRI in both genders. In total, 8.57% of the respondents were underweight, 60.95% were normal, 24.76% were overweight, and 5.71% were obese. About 6.0% males and 8.2% of females were underweight, while 14.5% of females and 35% of males were overweight. The mean waist circumferences were 86.1 ± 9.5 and 74.1 ± 11.5 cm for male and female respondents, respectively (P = 0.0001). About 99.0% of males and 94.5% of females had normal waist circumference, but about 1.0% of male and 5.5% of female were at high risk for abdominal obesity (P = 0.001). Also, the mean hip circumferences were 100 ± 7.2 and 98.7 ± 12.7 cm for male and female respondents respectively (P = 0.36). Moreover, The mean waist to hip ratios (WHR) were 0.86 ± 0.5 and 0.80 ± 0.6 cm for male and female respondents, respectively (P = 1.00). Also, About 100% of males and more than 92.7% of females had normal waist and waist hip ratio, but 6.4% of female were at risk. Nonparametric correlation, Spearman’s Rho test was used to determine the association between waist and hip circumferences and WHR with BMI. Significant associations were observed between waist circumference (Rho = 0.725, P = 0.01), hip circumference (Rho = 0.721, P = 0.01), WHR (Rho = 0.245, P = 0.01) and BMI, respectively (Table 3). 

**Table 2.** Associations Between Dietary Intakes and Body Weight Status (n = 210)

Energy/Nutrient	Male (n = 100)	Female (n = 110)	Daily Intake, %	Spearman’s r-value	P value
	DRI% ^a^	DRI%			
**Energy, Kcal**	62.14	88.11	-	-	-
**Protein, g **	135.90	111.22	13.3	0.68	0.001
**Carbohydrate, g **	182.52	176.83	49.4	0.85	0.001
**Fat, g **	ND	ND	37.3	0.87	0.001
**Fiber, g **	50.55	78.90	-	0.74	0.001
**Vitamin C, mg **	89.48	97.90	-	0.14	0.001
**Calcium, mg **	45.70	47.92	-	0.35	0.001

^a^ Abbreviatons: DRI, dietary intake reference; ND, Non Determined

**Table 3.** Association Between Anthropometrics Factor and Body Weight Status (BMI) (n = 210)

Variable	Body Weight Status	
	Spearman‘s r-value	P value
**Waist circumference, cm**	0.73	0.001
**Hip circumference, cm**	0.72	0.001
**Waist to hip ratio, cm**	0.25	0.001

## 5. Discussion

Many studies have shown that socio-demographic factors such as socio-economic factors, education and income, living arrangement and marital status were related to BMI (11, 12). Contrary to expectations, this study did not find any significant association between socio-demographic factors and BMI, except for gender, which was significantly correlated to BMI (X^2 ^= 3.78, P = 0.02). The mean BMI of females was lower than males (22.8 *vs.* 23.9). However, the energy intake of females was higher than males. Although the reason for this finding is not known, the lower BMI of the female students is in agreement with the findings of Brunt and Rhee (22) who studied 585 subjects in the USA, and Navia et al., who studied 150 subjects in Spain (23). In the current study there was no significant association between BMI categories and smoking. One reason might be that only 29.1% of the respondents smoked. It has been reported that substantial initiation of tobacco use happened before adulthood (24). This was in agreement with the findings of this study, since almost one-third of the respondents tried smoking before the age of 19. A previous study also reported that smoking incidence increases strongly between the ages of 15 and 21 years, and then stabilized (8). In the present study, there was no significant association between BMI and physical activity. This finding shows that respondents with higher BMI were involved with less physical activity. This finding was consistent with the results of the Malaysian adult nutrition survey that the prevalence of students with obesity was slightly lower among the persons with increased physical activity (25). The present study showed that carbohydrate and protein intakes of the participants were higher than DRI in both genders. Results also showed that Iranian students in general have increased their fat derived energy intake to 37.3%; while, energy intake from carbohydrate and protein decreased to 49.4% and 13.3%. These findings are to some extent comparable to a Malaysian study in which the intakes of carbohydrate, protein and fat were 59%, 14%, and 27%, respectively (26). Tarighat et al. have also reported that food intake of each Iranian citizen is about 40% more than required amounts. The average Iranians consume 40% more carbohydrates and 30% more fats, than what is needed (27). The percentage intake of fiber for both sexes was lower than DRI. Similarly, in a study it was found that students consumed less than 15 g of fiber every day (28). Also, in this study, the percentage intake of vitamin C and calcium of the male and female students were lower than the DRI. This finding was consistent with the results of another study which found that students consumed less vitamin C and calcium according to the DRI recommendation (29). Many studies have shown that the prevalence of overweight and obesity among youths and adults have increased to epidemic proportions, and health care costs related with these conditions have been raised considerably (30-32). Moreover, it has been reported that prevalence of abnormal weight and obesity are increasing (33). In sum, the present study revealed that about 45% of male students and more than 26% of Iranian female students at UPM were underweight or overweight. There were significant associations between BMI and gender, physical activity, dietary intake, body fat, waist and hip circumference, and WHR. The findings of this study can provide baseline data for designing nutritional programs to prevent nutritional disorders among Iranian postgraduate students in UPM. The researcher suggests focusing on the factors identified by this study for such programs. The cultural values and attitudes of foreign students might play an important role in the development of students’ dietary behaviors and eating disorders. The university authorities, especially the international office, should make a policy intended to ensure involvement of the UPM students in general in physical activities such as moderate and high-intensity walking. The university restaurants would be the next most likely targets of nutritional intervention plans.
